# Type 2 diabetes and reduced exercise tolerance: a review of the literature through an integrated physiology approach

**DOI:** 10.1186/s12933-020-01109-1

**Published:** 2020-09-05

**Authors:** Lorenzo Nesti, Nicola Riccardo Pugliese, Paolo Sciuto, Andrea Natali

**Affiliations:** 1 Metabolism, Nutrition and Atherosclerosis Lab, Dietologia Universitaria, Pisa, Italy; 2 Cardiopulmonary Test Lab, Department of Clinical and Experimental Medicine, University of Pisa, Via Savi 10, 56126 Pisa, Italy

**Keywords:** Type 2 diabetes mellitus, Cardiopulmonary exercise test, Exercise physiology, Exercise tolerance, Physical exercise, Exercise training, Aerobic capacity, Diabetic complications, Diabetic cardiomyopathy, Diabetic lung, Autonomic dysfunction, Diabetic myopathy

## Abstract

The association between type 2 diabetes mellitus (T2DM) and heart failure (HF) is well established. Early in the course of the diabetic disease, some degree of impaired exercise capacity (a powerful marker of health status with prognostic value) can be frequently highlighted in otherwise asymptomatic T2DM subjects. However, the literature is quite heterogeneous, and the underlying pathophysiologic mechanisms are far from clear. Imaging-cardiopulmonary exercise testing (CPET) is a non-invasive, provocative test providing a multi-variable assessment of pulmonary, cardiovascular, muscular, and cellular oxidative systems during exercise, capable of offering unique integrated pathophysiological information. With this review we aimed at defying the cardiorespiratory alterations revealed through imaging-CPET that appear specific of T2DM subjects without overt cardiovascular or pulmonary disease. In synthesis, there is compelling evidence indicating a reduction of peak workload, peak oxygen assumption, oxygen pulse, as well as ventilatory efficiency. On the contrary, evidence remains inconclusive about reduced peripheral oxygen extraction, impaired heart rate adjustment, and lower anaerobic threshold, compared to non-diabetic subjects. Based on the multiparametric evaluation provided by imaging-CPET, a dissection and a hierarchy of the underlying mechanisms can be obtained. Here we propose four possible integrated pathophysiological mechanisms, namely myocardiogenic, myogenic, vasculogenic and neurogenic. While each hypothesis alone can potentially explain the majority of the CPET alterations observed, seemingly different combinations exist in any given subject. Finally, a discussion on the effects -and on the physiological mechanisms-of physical activity and exercise training on oxygen uptake in T2DM subjects is also offered. The understanding of the early alterations in the cardiopulmonary response that are specific of T2DM would allow the early identification of those at a higher risk of developing HF and possibly help to understand the pathophysiological link between T2DM and HF.

## Introduction

Despite the substantial advances in the clinical management of both diabetes and cardiovascular diseases throughout the world, coronary artery disease and heart failure (HF) remain the leading causes of the shorter life expectancy observed in subjects with type 2 diabetes mellitus (T2DM) [1]. A reduced exercise capacity, expressed either by reduced achieved peak workload or peak oxygen uptake, is a powerful marker of impaired global health status as well as the hallmark of HF [2]. Indeed, reduced peak oxygen uptake bears a solid negative prognostic value both in the general population [3] and in subjects with, or at high risk of, cardiovascular diseases [4]. Asymptomatic T2DM subjects show both a reduced exercise tolerance [5] and an excess risk of developing HF, which are not entirely explained by known cardiovascular risk factors or coronary heart disease. Thus, an early diabetes-related cardiopulmonary impairment has been postulated [1].

Cardiopulmonary exercise test (CPET), by adding ventilation and gas exchange measurement to electrocardiography during a symptom-limited progressive exercise test, is a unique non-invasive protocol providing a multi-variable assessment of pulmonary, cardiovascular and muscular function during exercise. The addition of echocardiographic monitoring (“imaging-CPET”) can provide further information on the different aspects of cardiac function during exercise and their impact on effort intolerance, mainly used in HF patients [6]. In Table 1 are shown the variables directly measured during the test (primary) and their software modelling (secondary variables), divided according to the systems and organ functions that each of them can assess. The understanding of the early alterations in the cardiopulmonary response that are specific of T2DM subjects would allow the identification of those at a higher risk of developing HF and could orientate the therapeutic strategies as well. Unfortunately, despite the extensive literature available, which alterations are responsible for the reduced exercise tolerance in asymptomatic T2DM subjects without overt cardiovascular complications is still unclear and remains matter of debate.

**Table 1:** Variables evaluated during cardiopulmonary examination grouped by their physiological value

Physiological mechanism	Imaging-CPET primary variables	CPET secondary variables
Whole body	VO_2_, work	VO_2_ %
Cardiovascular system	HR, BP	VO_2_/HR
Cardiac electric activity	ECG morphology and rythm	Chronotropic insufficiency, impaired heart rate recovery
Ventilation	VE, V_T_	VE/VCO_2_, VE/VO_2_
Gas exchange	SpO_2_, PETCO_2_, PETO_2_	AT
Skeletal muscle	Borg scale	Δ(a − v)O_2_, AT
Metabolism	RER	AT
Systolic function	LVEF, S’, TAPSE	Systolic reserve
Diastolic function	E/A, E/e’, DT	Diastolic reserve

We indicated “primary” those directly evaluated by CPET hardware, while those derived by mathematical modeling are indicated as “secondary”

ECG, electrocardiography; HR, heart rate; BP, blood pressure; RER, respiratory exchange ratio; VO_2_, oxygen uptake; VE, minute ventilation; V_T_, tidal volume; VO_2_ %, percentage of peak oxygen uptake with respect to maximal theoretic VO_2_; AT, anaerobic threshold; RER, respiratory exchange ratio, E/A, E/A wave ratio; E/e’, E/e’ wave ratio; DT, deceleration time, TAPSE, tricuspid annular systolic excursion

With this review, by adopting an integrated physiology approach, we will try to highlight the cardiorespiratory alterations that are specific of non-complicated diabetes and discuss both their clinical relevance and, when possible, the underlying pathophysiology.

## Exercise capacity in type 2 diabetes and its clinical relevance

Several studies have evaluated the cardiopulmonary performance in response to physical exercise in T2DMM subjects consistently observing, with respect to normal subjects, a reduced maximal aerobic capacity measured either in metabolic equivalents (METs) or in peak oxygen uptake (VO_2peak_) [7–23]. This defect can be quantitatively appreciated from well-matched case-controls studies in subjects with T2DM and no clinically evident cardiovascular disease or overt diabetic complications, consistently appearing as a 20–30% reduction in VO_2peak_ in both adults [18, 24, 25] and adolescents [26, 27]. The determinants of the reduced aerobic capacity can be deduced from large cohort studies. In the LOOKAHEAD trial [5] (5783 overweight/obese subjects with T2DM) the major predictors were age, body mass index (BMI), female gender, non-white ethnicity, diabetes duration, insulin use and systolic blood pressure. On the contrary, one recent study found no differences in VO_2peak_ in well-controlled, relatively young T2DM subjects with a disease duration of less than 5 years and without clinical complications or comorbidities compared to carefully matched subjects without diabetes [28]. However, a reduced VO_2_/workload slope during graded exercise was observed in T2DM women with similar characteristics [18]. These observations suggest that whilst early, well-controlled T2DM may not show a significant reduction in maximal aerobic capacity, the earliest diabetes-related defect could be highlighted by a slight reduction in the efficiency with which oxygen is converted in external work. Unfortunately, this elegant analysis was not performed in other studies.

In brief, established T2DM (> 5 years duration) is associated with impaired cardiorespiratory fitness in the form of reduced exercise tolerance and a 20–30% reduction of VO_2peak_ with respect to gender and age-matched, sedentary, obese, non-diabetic subjects. Interestingly, neither the grade of habitual physical activity [9, 10] nor early lactic acidosis (T2DM patients do not show different blood lactate kinetics with respect to normal subjects [15]) justify this finding. Thus, we can conclude that it is inherent to the diabetic state and increases with disease duration. Of note, a 12% reduction in VO_2peak_ has been observed in first-degree relatives of T2DM subjects in comparison to accurately matched controls (also for habitual physical activity) suggesting a possible role of the genetic background [29]. On the other hand, the relationship observed between reduced VO_2peak_ and poor metabolic control [11, 13, 24, 30, 31] and presence of microvascular complications [12] clearly points toward a pathophysiological role for sustained hyperglycemia. The mechanism through which this can reduce VO_2peak_ is not clear, since variable degrees of functional impairment in each of the systems cooperating at oxygen delivery and utilization has been observed in T2DM individuals. We will outline those abnormalities and their relationship with VO_2peak_ in the following sections.

Notably, in T2DM subjects, reduced exercise capacity appears to be a predictor of all-cause mortality [32]. In 6213 men referred for clinical reasons to exercise test [33], 25% failed to achieve a submaximal exercise capacity (≥ 5 METs) and, when compared with those who achieved > 8 METs, their age-adjusted relative mortality risk from any cause was ~ 2.3 (95% CI 1.5–3.5). Noteworthy, in a large cohort of T2DM subjects (n = 2867), multivariate analysis demonstrated an inverse, almost linear association between achieved METs and all-cause mortality [34] that was more evident in the 50–65 years age group in whom +1METS was associated with a risk reduction of 27%, while it was 16% in those 65 + years. Cardiovascular mortality can also be predicted by poor exercise performance alike. In 609 T2DM individuals with negative ECG stress test, those with an exercise capacity > 85% of the predicted had a 48% lower chance of myocardial infarction, stroke, or death than those below or equal to 85% [35]. In a similar cohort of veterans [36], after an extensive adjustment for confounders (age, ethnicity, examination year, BMI, presence of cardiovascular disease, and cardiovascular risk factors), diabetic patients achieving < 5 METs at exercise testing were 70% ([13–254]) more likely to die for a cardiovascular event than those achieving at least 5 METs, the association being more robust and more graded for Caucasians than for African Americans [37].

## Determinants of reduced exercise capacity in type 2 diabetes

**Fig. 1:**
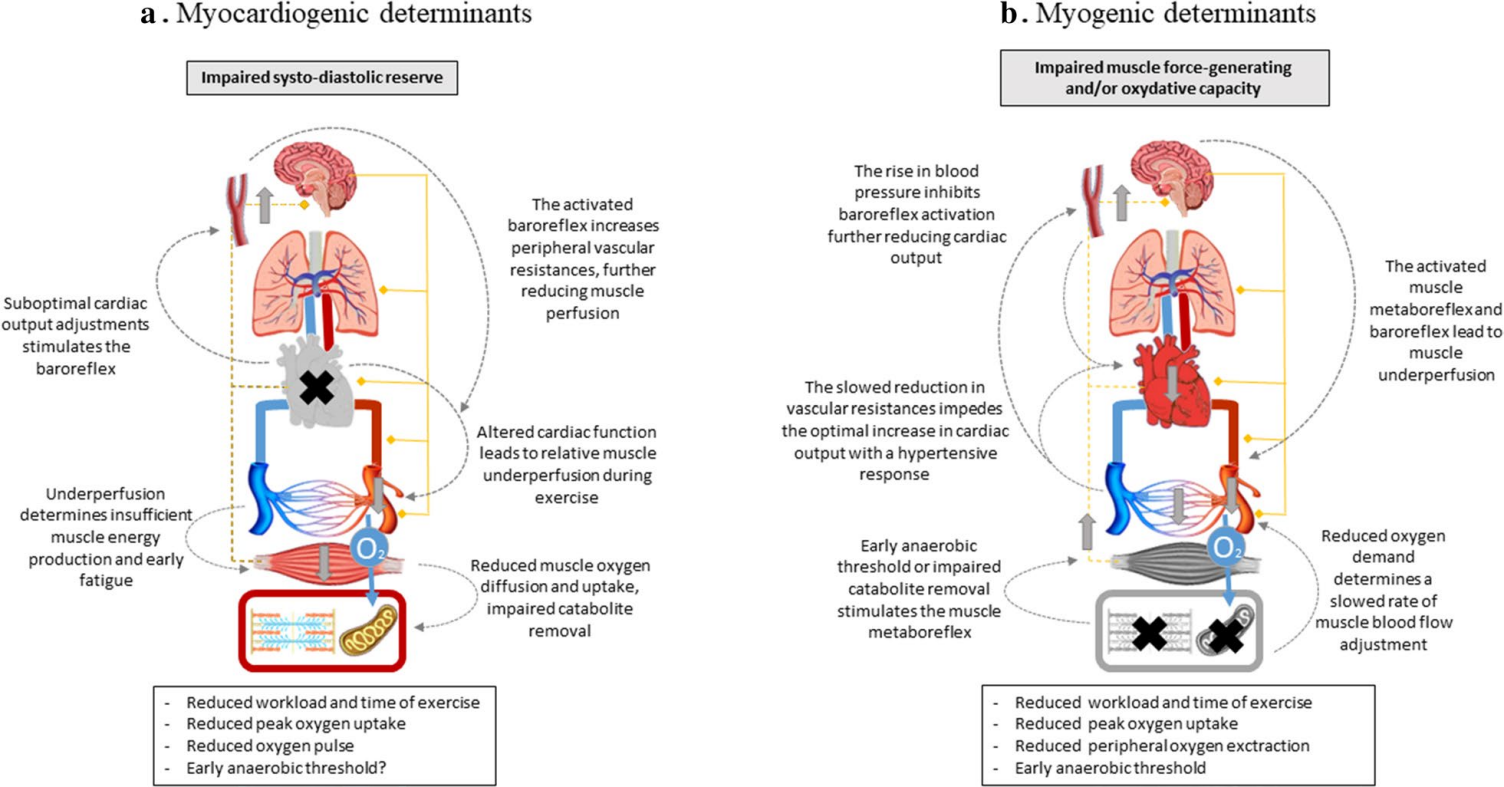
Myocardiogenic and skeletal myogenic determinants of reduced exercise tolerance in type 2 diabetes. Schematic representation of two pathophysiological hypotheses explaining the alterations observed in type 2 diabetes mellitus through cardiopulmonary exercise testing. Central and peripheral (efferent fibers to lungs, heart, conduit vessels, muscle arterioles are represented on the right, while afferent fibers of muscle metaboreflex and cardiac sympathetic reflex are represented on the left of the picture) nervous system, the baroreceptor, lungs, heart, conduit vessels, muscular vasculature, oxygen diffusion from capillaries to muscle, skeletal muscle and muscle cells are schematically represented. In each hypothesis, the organ/system that is primarily impaired is colored in grey and is marked with a black cross, whilst the other organs maintain their colors. Secondary involvement of other organs/systems is indicated with vertical grey arrows indicating stimulation (arrow facing up) or inhibition (arrow facing down) through known physiological mechanisms, which are represented as grey dotted lines with captions. On top of the figure, the grey text panel explains the primary involvement of the organ/system identified with the black cross and grey shadowing; the text panel beneath the figure shows a list of the altered cardiopulmonary exercise test variables observed in type 2 diabetes mellitus that may be explained by the pathophysiological hypothesis here shown. **a** *Myocardiogenic determinants*: an insufficient cardiac output adjustment reduces muscle perfusion, force production, and thus exercise tolerance. **b** *Skeletal myogenic determinants*: reduced skeletal muscle aerobic capacity or early fatigue can account for the reduced oxygen uptake, peripheral oxygen extraction, whilst a reduced cardiac output may be secondary to an enhanced baroreflex activation (see main text for full description)

**Fig. 2:**
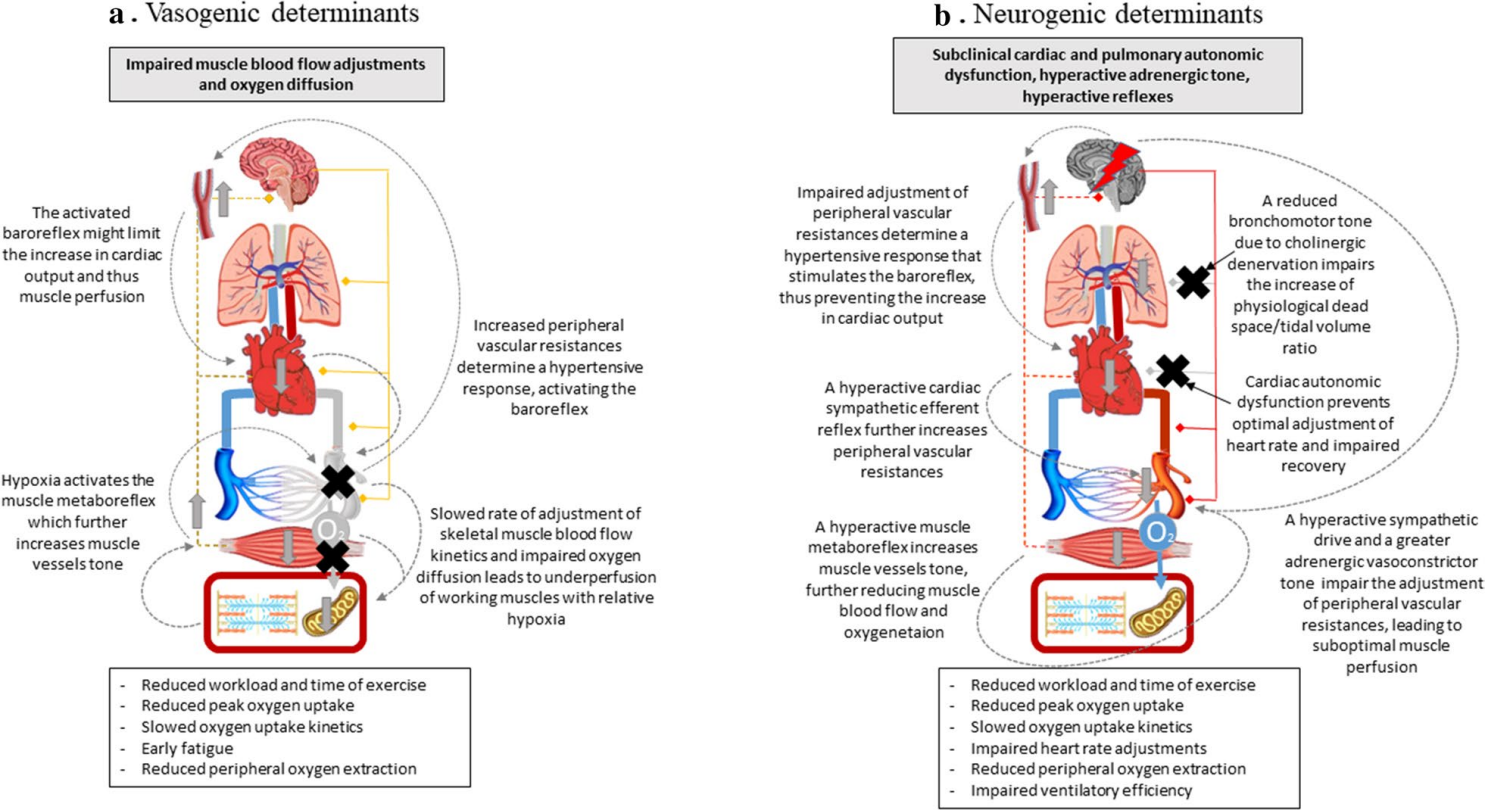
Vasogenic and neurogenic determinants of reduced exercise tolerance in type 2 diabetes. Schematic representation of two pathophysiological hypotheses explaining the alterations observed in type 2 diabetes mellitus through cardiopulmonary exercise testing. Central and peripheral (efferent fibers to lungs, heart, conduit vessels, muscle arterioles are represented on the right, while afferent fibers of muscle metaboreflex and cardiac sympathetic reflex are represented on the left of the picture) nervous system, the baroreceptor, lungs, heart, conduit vessels, muscular vasculature, oxygen diffusion from capillaries to muscle, skeletal muscle and muscle cells are schematically represented. In each hypothesis, the organ/system that is primarily impaired is colored in grey and is marked with a black cross, whilst the ones that are hyperactivated are marked with a red bolt and a red shadowing. Unaffected systems maintain their colors. Secondary involvement of other organs/systems is indicated with vertical grey arrows indicating stimulation (arrow facing up) or inhibition (arrow facing down) through known physiological mechanisms, which are represented as grey dotted lines with captions. On top of the figure, the grey text panel explains the primary involvement of the organ/system identified with the black cross and grey shadowing or the red bolt and red shadowing; the text panel beneath the figure shows a list of the altered cardiopulmonary exercise test variables observed in type 2 diabetes mellitus that may be explained by the pathophysiological hypothesis here shown. **a** *Vasogenic determinants*: impaired modulation of peripheral vascular resistances reduces muscle blood flow and oxygen diffusion, thus negatively affecting skeletal muscle performance, peripheral oxygen extraction and cardiac output through enhanced baroreflex and muscle metaboreflex activation. **b** *Neurogenic determinants*: altered autonomic tone and hyperactive muscle metaboreflex and cardiac sympathetic efferent reflex might account for impaired cardiac output, suboptimal vascular and pulmonary adjustments during exercise, eventually leading to reduced muscle perfusion and oxygen extraction (see main text for full description)

During exercise, optimal oxygen delivery and utilization imply a delicate interplay of multiple physiological functions, such as pulmonary ventilation, gas exchange, cardiac output, muscle blood distribution and diffusion, skeletal muscle aerobic and force-generating capacity, as well as fatigue perception. Since each of them has been singularly reported to be impaired in T2DM without having been measured simultaneously nor selectively manipulated in intervention clinical trials, it is hard to understand which one/s of these is/are to be considered as the main determinant/s of the observed impaired aerobic capacity. Based on the review of the available evidence, we will describe in detail the differences that T2DM patients show with respect to healthy subjects at CPET (Table 2) and discuss their pathophysiologic determinants highlighting the questions that are still open. This will be done according to four integrated pathophysiologic hypotheses, namely cardiogenic, myogenic, vasculogenic and neurogenic (Figs. 1, 2). Each hypothesis considers the impairment of a single organ as the first step in limiting exercise tolerance, with secondary involvement of the other systems through known pathophysiological interconnections.

**Table 2:** Physiological meaning of the alterations observed in type 2 diabetes, and clinical usefulness of various cardiopulmonary exercise test indices in the management of diabetic patients

CPET variable	Alterations seen in T2D	Pathophysiologic mechanisms	Clinical value
Heart rate kinetics	Chronotropic insufficiency, Attenuated heart rate recovery	Autonomic neuropathy	Prognostic
Exercise ECG	Eventual inducible ischemia	Epicardial vessel disease	Screening
Exercise capacity	Reduced	Physical deconditioning?	Unknown
Peak oxygen uptake (VO_2max_)	Reduced	Primary myocardial impairment	Prognostic
Oxygen kinetics	Slower	Primary myocardial impairment	Unknown
Oxygen pulse (VO_2_/HR)	Reduced	Primary myocardial impairment	Prognostic
Ventilatoy response (VE/VCO_2_)	Augmented	Primary ventilatory impairment	Unknown
Peripheral oxygen extraction (a-v O_2_)	Reduced	Skeletal muscle microcirculation impairment	Unknown
Anaerobic threshold (AT)	Unaltered, even in the presence of autonomic dysfunction	Aerobic/anaerobic metabolism	Unknown
VO_2_ at the anaerobic threshold (AT)	Possibly reduced	Unknown	Unknown

### Myocardiogenic determinants

Provided that oxygen extraction is maximal in the working muscle, the only way to deliver more oxygen to the bulk of active muscles is by increasing perfusion. During exercise, this is achieved through a rise in cardiac output that is coupled to a redistribution of blood away from less active tissues (skin and splanchnic vascular beds). The increase in cardiac output is obtained through a combination of an early increase in stroke volume (SV), plateauing approximately at 50% of peak effort, and a continuous increase of heart rate. In this paragraph we concentrate on SV and its determinants. CPET provides an indirect estimate of SV throughout a graded, progressive maximal exercise. As derived by Fick’s equation (Eq. 1, where CO is cardiac output; Δ(a − v)O_2_ is peripheral oxygen extraction; SV is stroke volume; HR is heart rate), oxygen pulse, calculated as VO_2_ divided by heart rate (VO_2_/HR), reflects the oxygen extraction capacity per heartbeat (Eq. 2):

1 $${\text{VO}}_{2} \left[ {{\raise0.7ex\hbox{$L$} \!\mathord{\left/ {\vphantom {L {min}}}\right.\kern-0pt} \!\lower0.7ex\hbox{${min}$}}} \right] = {\text{CO}} \cdot \Delta \left( {{\text{a}} - {\text{v}}} \right){\text{O}}_{2} = {\text{SV }} \cdot {\text{HR}} \cdot \Delta \left( {{\text{a}} - {\text{v}}} \right)O_{2}$$

2 $$\frac{{VO_{2} }}{HR}\; = \;SV \cdot \Delta \left( {{\text{a}} - {\text{v}}} \right)O_{2}$$

If we exclude other major factors influencing oxygen delivery (namely severe impaired pulmonary or cardiac function, anemia or hypoxia), systemic Δ(a − v)O_2_ during graded exercise has a constant and reproducible, almost linear pattern. Therefore, from Eq. 2, given the aforementioned assumptions, oxygen pulse provides an accurate [38] estimate of SV during exercise (Eq. 3):

3 $${\text{if}}\;\Delta \left( {{\text{a}} - {\text{v}}} \right){\text{O}}_{ 2} \approx {\text{ k}},{\text{ then}}\;\frac{{VO_{2} }}{HR}\; \approx {\text{ SV}}$$

The few studies reporting this variable all concordantly show that peak oxygen pulse appears reduced by 20–30% in diabetes [8, 15, 21] regardless of autonomic dysfunction, thus suggesting a primary cardiogenic limitation to exercise. Still, even with all the assumptions of the case, one should consider that peak oxygen pulse has a limited accuracy as a proxy for SV [39]. Thus, it is not possible to confirm a myocardial impairment based on this parameter alone. A comparison of the VO_2_/HR curves and their initial slope during graded exercise would offer a more reliable estimate of SV differences, but we could not find this data in T2DM without cardiac autonomic dysfunction.

In the last years, echocardiographic measures have been advantageously added to the CPET examination, providing useful information on heart morphological and functional data during incremental exercise. In asymptomatic T2DM, the use of standard echocardiography, tissue Doppler imaging, and speckle tracking technology during maximal exercise allowed the demonstration of a subtle myocardial dysfunction during exercise, which was not evident at rest [40]. In fact, despite normal baseline values, S’ (peak systolic annular) and E’ (early left ventricular filling phase) velocities of the mitral annulus measured through tissue Doppler were significantly reduced in patients with T2DM during exercise compared to controls, thus unmasking reduced systo-diastolic reserve [41–43]. Importantly, reduced VO_2peak_ was associated with S’ and E’ and also to cardiac magnetic resonance assessed myocardial fibrosis [44]. However, other studies did not observe impaired Doppler indices, but reported LV size as a predictor of reduced oxygen uptake [45]. Finally, cardiac index reserve, a variable expressed by the combination of chronotropic index and SV index reserve, appears related to VO_2max_ as well [46], thus strengthening the possibility of causal link between subclinical diabetic cardiac myopathy and reduced exercise capacity in T2DM. Unfortunately, associations do not provide information on the causal relationships, therefore it would be more informative to evaluate the full VO_2_/cardiac index curves and, more importantly, to verify whether VO_2peak_ can be normalized by pharmacologically improving the cardiac inotropic function. Interestingly, the cardiac index reserve was neither related to body weight nor metabolic control but was associated with subtle functional and structural myocardial abnormalities [40]. However, this is a highly debated topic, since other studies failed in finding associations between cardiac function and exercise tolerance [47]. The lessened increase in cardiac output would explain the reduced VO_2peak_ and VO_2_ kinetics and by preventing an adequate rise in blood pressure would enhance baroreflex stimulation, which, in turn, would impair perfusion of the exercising muscles and muscle force generation (Fig. 1a). Still, whether the insufficient increase in SV [48], in turn, depends on impaired left ventricle contractility or compliance remain to be clarified [19, 23, 49].

### Skeletal myogenic determinants

A reduced aerobic capacity might be secondary to reduced muscle aerobic power. However, together with the reduced peak workload, T2DM shows early development of fatigue, that is a perceived limitation of force-generating capacity that requires higher intensity of effort that might eventually reduce the exercise duration. This can be highlighted by an early appearance of exhaustion during exercise and in higher fatigue with respect to controls at any given workload, even when adjusted for the reduced VO_2peak_ [50, 51]. Still, the reasons for the decreased muscular exercise tolerance are far from being clear. One small study [25] found that insulin resistance, reduced muscle type 1 fiber content, and reduced muscle capillary density were the major contributor to the limited exercise capacity of patients with T2DM. In addition, impaired skeletal muscle mitochondrial function (measured as phosphocreatine recovery after exercise) has been reported in T2DM as well as in obese individuals [52]. Notably, in T2DM this appears more pronounced and correlates with glycated hemoglobin [53] suggesting that glucose toxicity, in addition to insulin resistance, might produce skeletal muscle dysfunction. Unfortunately, no study has simultaneously measured mitochondrial function and exercise capacity in T2DM. In any case, an impaired oxidative or force-generating muscle capacity might account for the reduced oxygen extraction and the slowed and reduced VO_2peak_ (Fig. 1b).

During CPET, an indirect way to evaluate the relevance of muscle aerobic capacity is through the analysis of the aerobic threshold (AT). As exercise intensity increases, muscle fibers initiate to undergo lactic (anaerobic) metabolism, with an increase in serum lactate concentration and hydrogen ions buffered by blood bicarbonate. This produces extra carbon dioxide (CO_2_) in adjunction to that derived by aerobic metabolism, thus reaching the curve breakpoint known as AT. The increase in VCO_2_ together with the subsequent decrease in blood pH stimulate a compensatory increase in the ventilatory (VE) curve, whose steep increase enables the non-invasive determination of the AT. The AT has been considered the gold standard along with VO_2peak_ for the evaluation of aerobic fitness and prescription of aerobic exercise [54]. Also, while VO_2peak_ is a direct measure of exercise capacity, a useful comprehensive estimate of aerobic efficiency can be obtained by measuring VO_2_ at AT, which also proved to predict cardiovascular and all-cause mortality in the general population [55]. Few studies have measured VO_2_ at AT in individuals with T2DM without overt cardiovascular disease through different methods including the ventilatory equivalents, heart rate and blood lactate. The available evidence is divided, showing either normal AT, even in the presence of autonomic dysfunction [17, 18, 20], or a reduced VO_2_ at AT [11, 16, 31, 56]. Interestingly, a reduced VO_2_ at AT was observed in T2DM patients with microalbuminuria but not in T2DM with normoalbuminuria [57], possibly suggesting that other factors unrelated to skeletal muscle biochemistry are at play. Clearly the evaluation of this parameter as a percent of VO_2peak_ would be more interesting; unfortunately, such information is still uncertain with one small study reporting a modest relative reduction [9] and other two no difference [14, 28] with respect to normal subjects.

Another indirect way to estimate skeletal muscle aerobic efficiency during CPET is calculating peripheral oxygen extraction (Δ(a − v)O_2_). During progressive maximal exercise, working muscles utilize progressively more oxygen to meet the increasing energy requests. Provided that pulmonary function is normal, the arterial oxygen content is usually maintained constant during exercise while the venous content is progressively reduced as a function of the extracting capacity of the working muscles. In contrast, during progressive maximal exercise, Δ(a − v)O_2_ increases until a plateau is reached when the maximal peripheral oxygen extraction has been attained. This value can be obtained by either direct measurement of blood oxygen or can be non-invasively estimated through the Fick’s equation (Eq. 4) from whole body oxygen uptake and cardiac output:

4 $$\Delta \left( {{\text{a}} - {\text{v}}} \right)O_{2} \; = \;\frac{{\dot{V}O_{2} }}{CO}$$

In the diabetic population, a high prevalence of HF with preserved ejection fraction (HFpEF) is observed [58]; since a reduced Δ(a − v)O_2_ using imaging-CPET has been previously demonstrated in patients with HFpEF in comparison to either controls or HF with reduced ejection fraction [59], one can hypothesize that it might play a role in T2DM alike. Indeed, only two studies have addressed this question reaching different conclusions [19, 49]. However, it should be noted that in the study by Baldi et al. cardiac output did not differ between T2DM and control, while Δ(a − v)O_2_ was reduced by 20% and correlated with VO_2peak_. The study showing no difference was conducted in a very small group of female adolescents.

### Vasogenic determinants

During skeletal muscle contraction, complex coordinated interplays exist between the cardiovascular system and the exercising muscle groups in order to optimize muscle perfusion to meet regional energy requests. Notably, peripheral vascular resistances are modulated in order to maximize the blood flow to the working muscles, since even a slightly reduced muscular oxygen partial pressure determines a reduced force production for a given motor neuron independently from a fatigue effect [60]. The mechanisms of reduced force production during mild hypoxia are numerous, encompassing either intracellular acidosis from anaerobic glucose breakdown or an increased ADP and inorganic phosphate-to-ATP ratio known to stimulate glycogenolysis producing additional intracellular acidosis [61]. These metabolic alterations lead to a reduced exercise intensity or, if the subject is willing to maintain the required exercise intensity, to a request for an increased motor neuron drive which is experienced as increased perceived exertion.

Vasodilation depends on the release of local mediators as well as on a preserved endothelial and smooth muscle cell function, which appear to be altered in T2DM. Their impact on VO_2peak_ emerges from correlations studies in subjects with T2DM [62] that are consistent with reduced muscle blood flow during exercise [63] and increased peripheral vascular resistance [64]. Slowed microvascular blood flow kinetics were reported in T2DM both at the beginning of exercise [65, 66] and during the steady-state [67, 68], with some heterogeneity among the studies. A slowed rate of adjustment of peripheral vascular resistance at the beginning of exercise could explain the slowed VO_2_ kinetics frequently observed in this population. A slower reduction in peripheral vascular resistance might also produce an increase in systemic blood pressure (as frequently observed in T2DM patients during CPET) possibly leading to a baroreflex-mediated limitation in cardiac output [69] (Fig. 2a). Several mechanisms can be recognised, namely feed-forward mechanisms (early adjustment of muscle blood flow by adenosine, potassium, nitric oxide) and feedback mechanisms (vasodilatory factors secondary to muscle metabolic demand and haemoglobin deoxygenation). In T2DM, while an impairment the feed-forward or muscle metabolic feedback mechanisms remains to be proved, reduced deoxygenation-related feedback mechanisms can be postulated. At rest, diabetic individuals show reduced ATP release from red blood cells in response to haemoglobin desaturation [70]. This mechanism appears impaired in T2DM, wherein it is activated through endothelial purinergic receptors that trigger NO-dependent and -independent arteriolar vasodilation, and is known to impact on muscle blood flow [71]. Also, several endothelial-related mechanisms of vasodilation (endothelial dysfunction) are known to be impaired in diabetes, with a substantial impact on muscle blood flow adjustments during both rest and exercise [67]. Oxidative stress has been consistently recognized as a key mediator of endothelial dysfunction in both acute [72] and chronic [73] hyperglycemia, having thus been considered amongst the main pathophysiological determinants of diabetic vascular complications. However, the relation between oxidative stress and exercise appears to be complex [74], and whether, and to what extent, this may impact on exercise tolerance in T2DM remains unclear. In a peculiar model of reversible elevated oxidative stress and endothelial dysfunction which is atrial fibrillation, cardioversion was able to improve exercise tolerance in non-diabetic subjects but resulted ineffective in subject with diabetes [21]. On the other hand, treatment with rosiglitazone was associated with an improvement of both VO_2peak_ and endothelial function (measured though brachial artery flow mediated dilatation) in a small group of subjects with T2DM. Despite the small improvement in VO_2peak_ (7%), the latter significantly correlated with the improvement in FMD [75]. Moreover, an exaggerated muscle metabolic reflex has been observed in T2DM (see paragraph 3.4) [76] a mechanisms that is known to produce further vasoconstriction in muscle arteries thus possibly aggravating regional blood flow impairment to the exercising muscles. In addition to muscle perfusion impairment, in T2DM an impaired oxygen diffusion from capillaries to muscle cells can be postulated. Both the increased oxygen affinity of glycated hemoglobin (displaying a left shift of the dissociation curve [77]) and the structural and functional capillaries rarefaction observed in T2DM muscles [78] might reduce oxygen diffusion from capillaries to muscle cells. To add further complexity to the scenario, it must be noted that alterations in muscle perfusion can also affect the muscle performance by inducing early fatigue independently from oxygen delivery, through an impaired catabolite removal [79].

### Cardiovascular neurogenic determinants

When a neural motor impulse is sent to a skeletal muscle, a parallel impulse is given to the cardiovascular centres in the brainstem in order to increase cardiac output and central blood pressure mainly through sympathetic activation. The second determinant of increased adrenergic sympathetic tone during exercise is the muscle metaboreceptor that is stimulated by muscle mechanical and metabolic activity. These two systems are further modulated by arterial and cardiopulmonary baroreflexes that allow a beat-per-beat optimal adjustment of systemic cardiovascular parameters in order to meet regional muscle metabolic demands. During exercise the systemic peripheral vascular bed normally undergoes sympathetic adrenergic vasoconstriction known as sympathetic restrain, which is meant to reduce blood flow to non-exercising tissues. In the working muscles, this is counterbalanced by local vasodilating mechanisms (functional sympatholysis) that allow muscular blood flow to adjust to meet the regional metabolic requests rapidly. In T2DM, several defects in the neural control of the cardiovascular responses to exercise have been observed.

A reduced sympathetic innervation of the heart, as observed in diabetic cardiac autonomic neuropathy [80], can reduce the response of the heart to the sympathetic stimuli and thus lead to several resting and exercising anomalies in heart rhythm, heart rate adjustments, and exercising cardiac output adjustments [14], possibly negatively impacting on VO_2peak_ and O_2_ kinetics. Moreover, a hyperactive sympathetic restrain can overcome the vasodilating capacities of the working muscles thus determining a reduced muscle blood flow [81], with detrimental consequences on muscle functions, increase peripheral resistances and thus limiting the increase in cardiac output though baroreflex overstimulation, and eventually also VO_2peak_ (Fig. 2b). There is preliminary evidence of both a hyperactive sympathetic adrenergic drive and a greater adrenergic vasoconstrictor response to noradrenaline in T2DM subjects at rest [82] that might blunt functional sympatholysis in skeletal muscle vessels. However, its role during exercise is currently unknown. The few available studies investigating heart rate kinetics in T2DM during exercise testing provide evidence that diabetes duration and metabolic control are implicated in the chronotropic response. In fact, a slower kinetics of adjustments of heart rate [18, 83] is more evident in older T2DM male with longer disease duration [84] or with suboptimal glycemic control [83]. On the contrary, well-controlled men with T2DM and with relatively short disease duration (< 5 years) do not show significant heart rate abnormalities with respect to control subjects [28]. In T2DM patients, alterations in time and frequency domains of heart rate variability at rest or in response to either exercise or other stimuli have been recognized as hallmarks of cardiac autonomic dysfunction [85], bearing a negative prognostic impact as highlighted in several major clinical trials such as ADVANCE, VADT, and ACCORD [86–88]. Noteworthy, parameters obtained during CPET examination allows the detection of earlier stages of cardiac autonomic dysfunction with respect to resting assessment [89]. In this setting, together with peak heart rate during exercise test, chronotropic incompetence and impaired heart rate recovery have a key physiological and clinical meaning, reflecting the dynamic balance between parasympathetic and sympathetic drives [90].

Chronotropic incompetence, defined as the inability to sufficiently (above 80% of maximal calculated) increase heart rate in response to increased activity, has been frequently observed in T2DM patients [91] wherein it appears related to exercise tolerance and adipose tissue mass [92]. Interestingly, it has been identified as one main factor associated with reduced exercise capacity after acute myocardial infarction in T2DM patients [93], and patients with T2DM and concomitant metabolic syndrome more frequently show chronotropic incompetence  [94]. Moreover, in a mixed population of diabetic outpatients, chronotropic incompetence predicts coronary artery disease [95, 96] and cardiovascular events and mortality [97]. Similarly, impaired heart rate recovery, that is a slower heart rate decrease within the first 1 or 2 min after the cessation of physical exercise [89], is highly prevalent in the asymptomatic T2DM population [98]. It was also linked to adverse cardiovascular outcomes and increased all-cause mortality in the diabetic population beyond traditional cardiovascular risk factors [97, 99]. On these bases, an exercise test for the detection of abnormal heart rate recovery has been proposed for routine cardiovascular screening [100]. Interestingly, slower heart rate recovery appears strongly coupled with reduced physical fitness and with chronotropic incompetence during exercise [98, 101]. Indeed, heart rate recovery was significantly ameliorated after a 12 weeks aerobic exercise training in 30 T2DM Chinese men [102]. Provided that aerobic training is known to improve autonomic function in cardiac neuropathy, this might support the hypothesis that altered peak and post-exercise heart rate in T2DM derives mainly from subclinical autonomic dysfunction. However, further studies are necessary to confirm it.

Mechanical and metabolic stimuli produced by contracting muscle stimulate receptors and channels on the peripheral endings of thinly myelinated group III (Aδ-fibers) and unmyelinated group IV (C-fibers) skeletal muscle afferents that are sensitive to mechanical and metabolic (lactic acid, bradykinin, arachidonic acid, ATP) stimuli generated in the exercising muscles. The muscle afferents are integrated into the brainstem and determine the activation of sympathetic responses that increase cardiac output and peripheral vascular resistance. This reflex arch, known as muscle metaboreflex, has been demonstrated hyperactivated in T2DM [76], possibly accounting for a hypertensive blood pressure response and an impaired muscular performance secondary to reduced muscle blood flow. Interestingly, the hyperactivation of muscle metaboreflex efferents appears to be significantly correlated with diabetic disease severity [76] suggesting a pathophysiologic role for sustained hyperglycaemia and also providing a mechanistic explanation for the attenuated alterations seen in the well-controlled early T2DM.

Similarly, the cardiac sympathetic afferent reflex is a positive sympathoexcitatory neural feedback activated by a relative myocardial underperfusion (the metabolic stimuli are reactive oxygen species, acidosis, adenosine, endothelin-1, all observed to be increased in the diabetic heart [103]) that results in increases in systemic blood pressure, heart rate and myocardial contractility. This was found enhanced in T2DM animals [104], whilst in humans the exaggerated blood pressure response and the increased systemic catecholamines seen during exercise [105] might further support the hypothesis of a role for a hyperactive cardiac sympathetic afferent reflex in T2DM. However, its role in affecting cardiopulmonary performance is still to be proved.

### Pulmonary neurogenic determinants

Pulmonary autonomic neuropathy might explain the alterations in ventilation and respiratory gases frequently seen in T2DM patients [15–17]. The steepness with which pulmonary ventilation (VE) rises in relation to carbon dioxide production (VCO_2_) during progressive exercise, known as ventilatory equivalent for CO_2_ or more improperly “ventilatory efficiency”, is the most used index of pulmonary function measured during CPET. The slope of the relationship between ventilation and CO_2_ output (VE/VCO_2_ slope) is linear over a wide range during exercise and appears raised in several pulmonary and cardiac diseases, highlighting an inappropriate (hyper)ventilatory response to VCO_2_. It is worth noting that a raised VE/VCO_2_ slope is associated with adverse prognosis [106]. Even if not confirmed by all studies [15, 21, 107], an increased VE/VCO_2_ slope has been found in both uncomplicated T2DM and in T2DM with cardiac autonomic dysfunction, in the latter appearing even more pronounced than in uncomplicated T2DM [15–17]. Thus, T2DM subjects appear to hyperventilate in relation to CO_2_ production during exercise with respect to normal subjects. The pathophysiological determinants are to be searched in the modified alveolar equation:

5 $$\dot{V}{\text{E }} = 863 \cdot \frac{{\dot{V}CO_{2} }}{{PaCO_{2} \cdot \left[ {1 {-} \frac{{V_{D} }}{{V_{T} }}} \right]}}$$

where PaCO_2_ is the arterial partial pressure of carbon dioxide (in CPET the end-tidal CO_2_ partial pressure, that is P_ET_CO_2_, is used as an estimate of PaCO_2_), and V_D_/V_T_ is the physiological dead space (V_D_)/tidal volume (V_T_) ratio [108]. From Eq. 5 we can deduce the determinants of ventilatory efficiency:

6 $$\frac{{\dot{V}{\text{E}}}}{{\dot{V}CO_{2} }} = \frac{863}{{PaCO_{2} \cdot \left[ {1 {-} \frac{{V_{D} }}{{V_{T} }}} \right]}}$$

As we can see from Eq. 6, an increased VE/VCO_2_ slope might derive from either a reduced PaCO_2_ or an increased V_D_ with respect to V_T_. In healthy individuals, it is known that the PaCO_2_ is maintained constant during exercise thanks to the appropriate increase in ventilation mainly stimulated by increased CO_2_ output, while the V_D_ is progressively reduced due to progressive recruitment of non-ventilating alveoli reducing the V_D_ and increasing the V_T_ [108]. Available evidence suggests that both parameters are altered in T2DM. In fact, a reduced rate of decrease in V_D_/V_T_ during exercise was observed in comparison to normal subjects, displaying higher T_D_ values at similar levels of ventilatory response [15]. Several mechanisms have been proposed, especially increased airways stiffness secondary to decreased bronchomotor tone due to bronchial cholinergic denervation (Fig. 2b), possibly accounting for the steeper VE/VCO_2_ slope found in T2DM subjects with autonomic dysfunction with respect to T2DM patients free from this complication. Further, a slightly reduced P_ET_CO_2_ at peak exercise was observed, together with an increased neuromuscular drive as measured through the pressure generated at the mouth during the first 0.1 s of inspiration (P_0.1_) [109]. The reasons for this are less easy to explain. In fact, a reduced P_ET_CO_2_ might be either the primary drive of hyperventilation or its consequence. In the first hypothesis, an altered central control of breathing must be implied, as revealed by the fact that in T2DM CO_2_ output can modulate the cerebral reactivity to CO_2_ [109]. The presence of diabetic neuropathy can further influence the cerebral reactivity to CO_2_ proportionately to the severity of the autonomic damage with the efferent sympathetic nervous system being involved in the increased respiratory drive seen in T2DM [107]. As a matter of fact, the increased ventilatory drive in T2DM might be secondary either to an altered CO_2_ set point or to a disproportionate central reactivity to CO_2_, or even to an impaired efferent sympathetic drive. However, it is not clear whether diabetes per se might be accountable for this alteration, or if some grade of autonomic derangement is necessary. In opposition, the reduced P_ET_CO_2_ can also be the consequence, and not the cause, of disproportionate ventilation. This might be a result of the reduced decrease in V_D_/V_T_. Moreover, the observed increased P_0.1_ might depend on mechanical constraints of the thoracic wall-pleura-lung system that are not evident at rest but become increasingly important with increased ventilatory requirements leading to relative (to VCO_2_) hyperventilation as response to increasingly energy-demanding, uncomfortable expansion of the chest wall. Intriguingly, also pulmonary ventilation (VE) appears dependent on glycemic control [30] with unknown mechanisms, possibly reinforcing the hypothesis of a structural and/or functional impairment that has been named “diabetic lung” [110]. However, it must be noted that the exercise induced VE response to PaCO_2_ is further modulated by a complex interplay of pulmonary vagal stretch receptors, peripheral and central chemoreceptors, baroreceptors, as well as ergoreceptors in skeletal muscles, that are difficult to isolate or completely neglect. Thus, we cannot exclude the dependence of the observed alterations in the ventilatory response from other mechanisms capable of altering the set point of PaCO_2_ regulation.

## Effect of exercise training

As a means to general health, physical exercise has a long and storied history and -as intensely suggested by all the major guidelines [111]-remains the cornerstone of lifestyle modification as the first step of non-pharmacological therapeutic strategies for T2DM. Indeed, it is widely known that whilst a sedentary lifestyle is a major risk factor for T2DM and its complications [112], maintaining an appropriate level of physical activity proves to be protective for both incident T2DM and cardiovascular disease [113]. In this setting, in addition to improving both short-term and long-term glycaemic control in T2DM [114], exercise training shows several direct beneficial effects on various organs and systems in T2DM subjects. A 16 weeks-long progressive training programme reduced abdominal fat, fasting glycemia and insulin sensitivity beyond weight loss [115]. This might be related to either an increased skeletal muscle glucose uptake with both insulin dependent and independent mechanisms [116], or to an anti-inflammatory effect on adipose tissue. In fact, long-term (12 months), resistance + aerobic exercise training has been demonstrated to suppress cytokine production through reduced inflammatory cell infiltration of adipose tissue and improved adipocyte function [117]. Also, the autonomic tone was proved to ameliorate after 12 weeks of personalised physical training (both aerobic and resistance exercise) [102], as well as endothelial function [118], intima-media thickness, and arterial stiffness [119]. Notably, also several echocardiographic parameters can be improved with exercise training [120]. Despite these positive adaptations, however, scepticism remains surrounding the effect of exercise training on oxygen uptake and prognosis in the T2DM population. Indeed, recently, an intensive lifestyle intervention proved to be ineffective in a large cohort of T2DM patients in affecting the risk of HF development [121], wherein a higher baseline cardiorespiratory fitness, the decrease in body weight, and the improvement in oxygen uptake were all associated with a small reduction in HF risk.

Structured programs of exercise training demonstrated to improve VO_2peak_ by approximately 10% in subjects with T2DM [122]. A metanalysis on 9 studies published in 2003 yields a figure of + 11%, but also a standard deviation of 11%, which indicates a surprisingly elevated interindividual variability. This aspect has been addressed by a large study by Pandley et al. [123], showing that up to 43% of T2DM patients undergoing structured exercise training, experience no improvement in VO_2peak_. Noteworthy, only 37% of them had a clinically meaningful response (ΔVO_2peak_ ≥ 5%). This is somewhat unexpected, since -on average- patients with T2DM are less physically active than non-diabetic subjects, and larger responses are usually observed in sedentary subjects undergoing physical exercise training than in already physically active individuals. In a group of only women, T2DM was associated with lower baseline VO_2peak_ and greater improvements in response to training, but -notably- they did not achieve the same level of fitness as the non-diabetic controls did while undergoing the same programme [124]. Overall, the balance of evidence appears to suggest that exercise training is not particularly effective in increasing VO_2peak_ in T2DM. Besides, these observations also indirectly suggest that muscle deconditioning does not entirely explain the lower VO_2peak_ of T2DM subjects. Seemingly, obesity -which shows a high prevalence in T2DM population- seem not to account for this effect either, since its role appears to be blunted across all BMI groups when cardiorespiratory fitness is considered [125]. Although a randomized controlled trial directly addressing this question is lacking, few indirect evidences provide further support to this hypothesis. An accurate study in middle aged non-diabetic subjects, estimated an impact of exercise training on VO_2peak_ that is slightly larger in quantitative terms (15%) and also more predictable (Standard Deviation: 8%) than what is generally reported in T2DM subjects [126], but still confirming the reduced response of T2DM patients to exercise training. Interestingly, the same observations seem to hold true also in diabetic subject with HF, wherein a training programme produced a smaller mean increase in VO_2peak_ than in non-diabetic counterparts (0.5 ± 2.4 vs 0.9 ± 2.6 mL kg − 1 min − 1; P = 0.03) [127].

In this scenario, three main sources of variability can be identified: namely, the type, the intensity, and the duration of the training performed. Indeed, in a large cohort of T2DM subjects, only the combination of resistance and aerobic training was able to increase VO_2peak_ by 5%, whilst neither of the same interventions alone resulted effective [128]. Secondarily, in a recent study the high intensity programme was able to produce a 20% increase of VO_2peak_, whereas moderate intensity resulted ineffective [129]. Additionally, high intensity interval training was proved more effective than moderate-intensity continuous training in improving cardiopulmonary performance in prediabetes and T2DM [130], suggesting that higher intensities are required to achieve significative results. However, since moderate intensity continuous training + resistance training increased oxygen uptake in T2DM patients after one year of supervised training -while high intensity interval training did not after the same follow-up- [131], the duration of the training performed should also be considered as a key factor.

The molecular background for the impaired response to exercise training in T2DM could depend on the toxic effect of diabetes on mitochondrial functions. Indeed, the effect of training on cellular respiration appears to be blunted in subjects with T2DM: even in patients with early-onset T2DM, abnormalities in the exercise-dependent pathway that regulates the expression of PGC-1alpha and Mfn2 have been demonstrated [132]. Proteomic studies seem to confirm this hypothesis, showing no change in protein expression in T2DM skeletal muscle in response to exercise, specifically at the site of the mitochondrial electron transport chain [133]. In addition, diabetes-related endothelial dysfunction might also explain the reduced increase in cardiorespiratory fitness after exercise training. Whilst the improvement in endothelial function is associated with the increased cardiorespiratory fitness in both T2DM subjects and the general population [134], the increase appears smaller in the first ones [118, 134]. Another explanation for the reduced sensitivity of VO_2peak_ to physical exercise in T2DM could be related to an interference of glucose lowering drugs with mitochondria. As recently shown, the ongoing treatment with metformin almost completely prevented the increase in VO_2peak_ induced by exercise training in T2D patients, as well as the exercise‐mediated increase in skeletal muscle mitochondrial respiration [135]. However, since improvements in VO_2peak_ are comparable between T2D offspring and control subjects (+ 15%), it is unlikely to postulate that a different skeletal muscle mitochondria response to exercise is present before the onset of diabetes [136]. The quantitative effect of exercise training on exercise tolerance in T2DM, as well as the prognostic value in terms of HF incidence and the pathophysiological mechanisms implicated, remain elusive. Further studies are intensely needed.

## Future directions

**Fig. 3:**
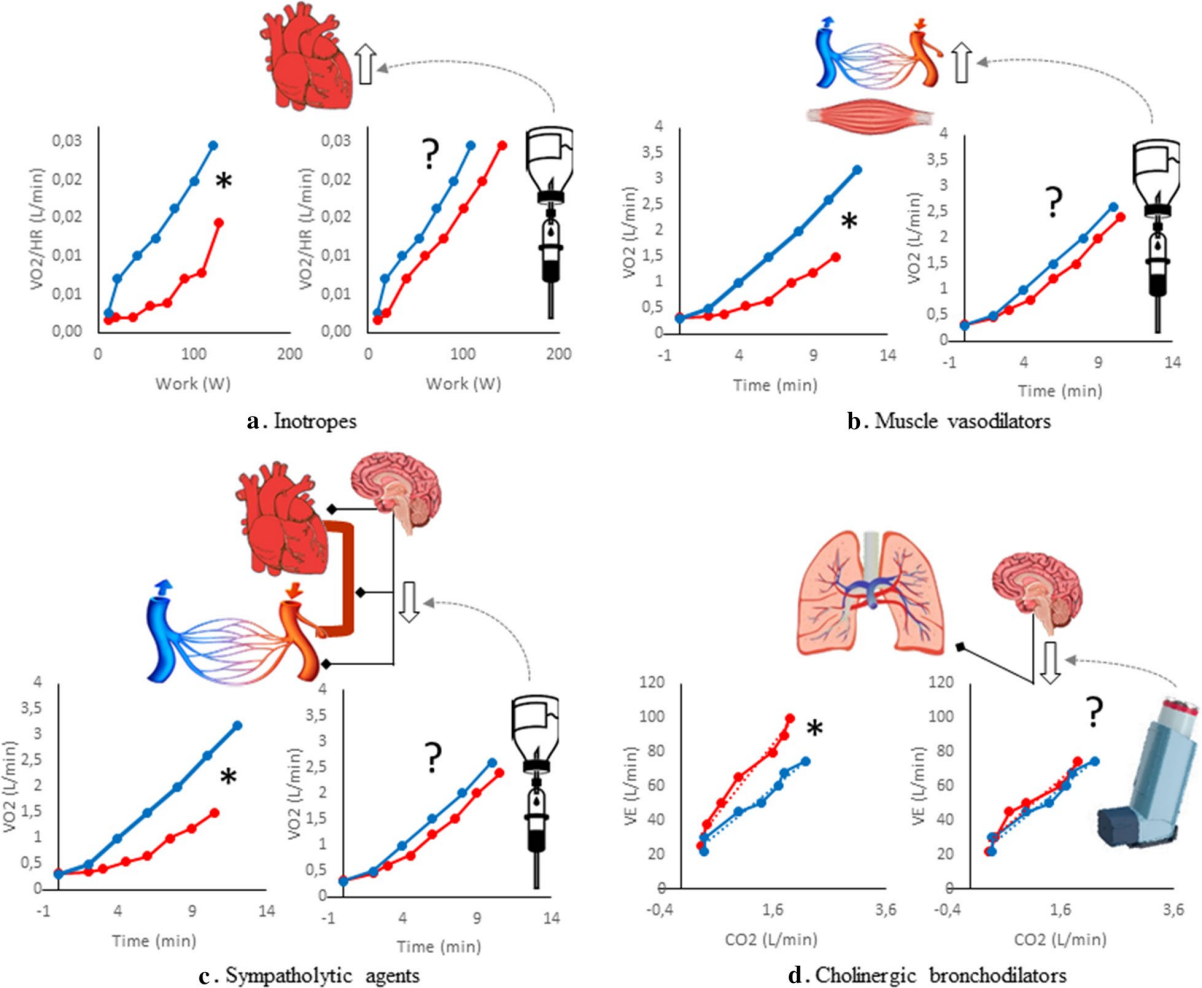
Proposed intervention trials. Four proposals for pharmacological interventions that could help address the relative importance of individual organ contributions to exercise intolerance in type 2 diabetes are here schematized. Grey vertical arrows indicate either enhancement (arrows facing up) or inhibition (arrows facing down) by the pharmacological treatment on the target organ. The red lines represent diabetic subjects and the blue ones the controls. The cardiopulmonary exercise test variables that investigate the organs implicated (schematized on top of each graphic) are qualitatively showed in the graphic on the left side of each subsection, while on the right there is the effect that we could expect if the organ schematized is the principal responsible for the impairment in the variable outlined (the gap between diabetic and non-diabetic patients is reduced or abolished). The Asterix (*) indicates the difference between these two populations, based on literature review. **a** The use of positive inotropes -like dobutamine- could confirm the weight of a reduced stroke work on oxygen uptake per heartbeat if a normalization is observed. **b** Muscle perfusion enhancers -like dipyridamole- could highlight the contribution of skeletal muscle vasculature adjustments by ameliorating oxygen uptake. **c** By reducing a hyperactive sympathetic restrain, sympathetic inhibitors might ameliorate cardiovascular adjustments and thus oxygen assumption, confirming the role of autonomic imbalance in exercise intolerance. **d** Anticholinergic bronchodilators could normalize “ventilatory efficiency” by facilitating the reduction of physiological dead space if the latter is due to bronchial cholinergic hyperactivation. VO_2_, oxygen uptake, VO_2_/HR, oxygen pulse; VE, ventilation

Whilst the evidence of reduced exercise capacity and tolerance in T2DM patients is quite homogeneous, the same cannot be said for its physiological determinants. Given that exercise intolerance is the hallmark of HF, and that the pathophysiological link between T2DM and HFpEF is still an intensely discussed topic, a better understanding of the determinants of the early alterations that prelude to overt HF in uncomplicated T2DM could be of great value. First, a more detailed description of the cardiopulmonary performance of T2DM subjects with exercise intolerance, in comparison to those with normal exercise tolerance, would establish which is/are the major determinant/s and allow the design of focused intervention studies. Second, well-designed intervention trials using imaging-CPET could help confirm or weaken the importance of each of the four hypotheses that here we propose. Based on the present work, we identified few intervention trials that could help dissect the relevance of each physiological system in exercise intolerance in T2DM. The simultaneous comparison of peak cardiac output and peak Δ(a − v)O_2_ according to the achieved VO_2peak_ in a large cohort would be required to establish whether the most important defect is in the heart or in the skeletal muscle. Conversely, being the role of vasodilatation in limiting exercise tolerance in diabetes extremely difficult to be established, only a well-designed interventional study with exercise skeletal muscle perfusion enhancers (like dipyridamole or theophylline) in subjects with low exercise tolerance could test the hypothesis. A trial with a low dose of a sympatholytic agent like clonidine or phentolamine could provide this information since it did not limit the time to exhaustion in normal subjects, while having minor systemic effects [137]. Furthermore, the role of a possible pulmonary neurogenic limitation to the decrease in V_D_/V_T_ might be highlighted by the change of VE/VCO_2_ slope in response to bronchodilators acting on cholinergic receptors (responsible for the bronchomotor tone and its adjustments during graded exercise). The questions that remain open and the experiments required to address them are schematized in Fig. 3. Imaging-CPET might also be used to dissect the mechanisms of improvement of VO_2peak_ after different types, intensities, and duration of exercise training. In this setting, it might play a pivotal role in shedding light on the physiological mechanisms (heart vs lung vs skeletal muscle vs blood vessels vs nervous system) that underpin the beneficial effects of physical exercise in T2DM. Furthermore, imaging-CPET might also be considered for rehabilitation programmes in T2DM patients. Currently, T2DM per se is not an official indication for performing CPET, a population in which the 6 min walking test is often seen as the first choice-since it is validated against exercise test [138]-yet quite uninformative. On the contrary, CPET allows a precise estimate of oxygen dynamics (which is also preserved in patients with coronary artery disease, appearing safe and very informative-even at very reduced workloads [139]), together with a multivariable cardiopulmonary, muscular and metabolic assessment; therefore, it might be envisaged not only for the assessment of cardiorespiratory fitness, but also for HF risk stratification and rehabilitation prescription and surveillance alike.

## Concluding remarks

There is solid evidence indicating reduced exercise tolerance in T2DM patients free from overt cardiovascular and pulmonary disease in the form of reduced peak workload, peak oxygen assumption, oxygen pulse, as well as reduced “ventilatory efficiency”. Based on the multiparametric evaluation provided by imaging-CPET, four pathophysiological determinants can explain the observed cardiopulmonary alterations, namely (1) myocardiogenic (inadequate cardiac output adjustments); (2) skeletal myogenic (reduced force-generating capacity and early fatigue); (3) vasogenic (suboptimal perfusion and oxygenation of working muscles); and (4) neurogenic (impaired neural control of cardiovascular and pulmonary adjustments during exercise). While each hypothesis alone can potentially explain the majority of the CPET alterations observed, seemingly different combinations exist—depending largely on diabetes duration and degree of metabolic control—in any given subject. Well-designed intervention studies using the multivariable approach offered by imaging-CPET might allow a better dissection of the role that each of the four hypotheses here proposed can have in determining an impaired exercise capacity. Imaging-CPET might also be a key technique for the dissection of the effects of physical exercise in T2DM. Imaging-CPET can be envisaged as a key tool for cardiovascular risk stratification, prognostication and rehabilitation in T2DM, capable of bringing pathophysiological insights into exercise intolerance and -possibly- the progression to overt HF.

## Limitations

The aim of our work was not to produce a systematic review, but a comment on the pathophysiology of exercise intolerance in T2DM; thus, we did not use the PRISMA guidelines. The methodology used for literature review was a search on Pubmed central with the keywords “type 2 diabetes” and a union of many keywords for exercise intolerance (e.g.: exercise tolerance, aerobic fitness, aerobic capacity, cardiopulmonary function, oxygen uptake, oxygen consumption, cardiopulmonary characterization, exercise capacity, exercise tolerance, exercise intolerance, diabetic cardiomyopathy, pulmonary function, lung function, exercise echocardiography, exercise imaging) and then looking for similar results among the suggested papers and through the references, aiming at a narrative review instead of a systematic review/metanalysis. We acknowledge that a physiology/narrative review does not have the same accuracy as a systematic review, thus we recognize it as a major limitation.

## Data Availability

Not applicable.

## Abbreviations

AT: Aerobic threshold
BMI: Body mass index
CO: Cardiac output
CPET: Cardiopulmonary exercise test
E’ Early: left ventricular filling phase velocity
HF: Heart failure
HFpEF: Heart failure with preserved ejection fraction
HR: Heart rate
METs: Metabolic equivalents
P_0.1_: Pressure generated at the mouth during the first 0.1 s of inspiration
PaCO_2_: Arterial partial pressure of carbon dioxide
PETCO_2_: End-tidal carbon dioxide partial pressure
S’: Peak systolic annular velocity
SV: Stroke volume
T2DM: Type 2 diabetes mellitus
VCO_2_: Carbon dioxide emission measured at the mouth
V_D_/V_T_: Physiological dead space/tidal volume ratio
VE: Pulmonary minute ventilation
VE/VCO_2_: ventilatory equivalent for carbon dioxide or “ventilatory efficiency”
VO_2_: Oxygen uptake measured at the mouth
VO_2_/HR: Oxygen pulse
VO_2max_: Maximal theorical oxygen uptake
VO_2peak_: Peak oxygen uptake
Δ(a − v)O_2_: Peripheral oxygen extraction
